# Porcine epidemic diarrhoea: new insights into an old disease

**DOI:** 10.1186/s40813-015-0007-9

**Published:** 2015-09-29

**Authors:** Ana Carvajal, Héctor Argüello, F. Javier Martínez-Lobo, Sara Costillas, Rubén Miranda, Pedro J. G. de Nova, Pedro Rubio

**Affiliations:** 1 Department of Animal Health, University of León, Campus de Vegazana. 24071, León, Spain; 2 Food Safety Department, Teagasc Food Research Centre, Dublin, Ireland; 3 Department of Animal Health, Complutense University of Madrid, Madrid, Spain

**Keywords:** Porcine epidemic diarrhoea virus, Coronavirus, Swine

## Abstract

Porcine epidemic diarrhea (PED) is an enteric disease in swine caused by an alphacoronavirus. It affects swine of all ages causing acute diarrhoea and can lead to severe dehydration and death in suckling piglets. Being recognized for the first time in Europe and Asia during the seventies and the eighties, respectively, it has remained a relevant cause of diarrhea outbreaks in Asia for years and to the present. It has become a major concern in swine production since 2013 when the virus was detected for first time in the USA and in other American countries causing a high number of pig deaths and significant economic losses. The present review aims at approaching the reader to the state of the art of PED giving answer to some of the most recent questions which have arisen related to this disease.

## Introduction

Porcine epidemic diarrhoea (PED) is a highly contagious infectious disease caused by a coronavirus, porcine epidemic diarrhoea virus (PEDV). It causes acute and watery diarrhoea in pigs of all ages although the most severe signs are reported in piglets less than two weeks old, in which diarrhoea leads to severe dehydration and is associated with mortalities which can reach up to 100 % in affected litters.

The first clinical description of PED occurred in the UK and Belgium in the early seventies. However, it was not until 1978 when the etiological agent of these diarrhoeal outbreaks, a new coronavirus, was identified [1, 2]. Soon afterwards, studies by the research group led by Professor Pensaert in Ghent, Belgium, demonstrated that there were no specific antibodies against PEDV in sera collected from sows prior to 1971, confirming that PEDV was a new virus in the European swine population. Up to now, there is no information available on the potential origin of this virus.

## Review

### Distribution

After its first description in UK and Belgium, PEDV spread throughout European countries causing diarrhoeal outbreaks in a relevant number of pig herds [3–5]. PEDV or specific antibodies against PEDV were reported in several European countries (Belgium, the UK, the Netherlands, Germany, Hungary, Bulgaria, France, Switzerland and Spain) in the seventies and the eighties [3–5]. Mortality in piglets less than two weeks old varied from 0 to 100 %, but it was usually lower than that described in outbreaks of diarrhoea caused by transmissible gastroenteritis virus (TGEV) which is another porcine coronavirus classically recognized as a cause of diarrhoea disease in swine. However, for unknown reasons, PED outbreaks markedly decreased in the nineties and in subsequent years in Europe. Isolated outbreaks associated with low mortality in piglets were reported in some countries, i.e. Spain [6], Hungary [7], the UK [8] or the Czech Republic [9, 10]. The only well-documented PED epidemic over the last 10 years in Europe occurred during the winter of 2005–2006 in northern Italy. On average, pre-weaning mortality raised from 8.3 to 11.9 %, peaking at 34.5 % in one particular farm [11]. Recent PED outbreaks have been reported in Germany [12–14], Italy [15, 16], the Netherlands [17], Belgium [18], France [19], the Ukraine [20] and other European countries (unpublished data).

In contrast to the situation in Europe, PEDV has remained as a major cause of diarrhoea outbreaks on swine farms in Asia for over 30 years. Viral-like diarrhoea outbreaks were reported on pig farms in Shanghai, China in 1973 and despite the fact that TGEV and other known enteropathogenic agents were ruled out, the aetiology could not be determined. PEDV was firstly demonstrated in the area in 1983 in China [21] and Japan [22]. In the nineties the virus spread to neighbouring countries such as Korea [23], the Philippines and Thailand [24]. Later on, it was reported in Taiwan in 2007 [25] and Vietnam in 2009 [26]. Due to its relevance in this area, attenuated or killed vaccines, which confer partial protection against PEDV, have been used in several Asian countries. These vaccines have been used in China since 1995 and also introduced in Japan in 1997, South Korea in 2004 and the Philippines in 2011 [27]. It is relevant to point out that in October 2010, a large-scale and severe PED outbreak was reported in several provinces in southern China and spread to other provinces within this country as well as to other neighbouring countries [28, 29]. The outbreak caused high mortality among suckling piglets, between 50 and 90 % and given the fact that China was clearly not a naïve country regarding PEDV infection, it was proposed that probably a new variant of PEDV with a higher virulence was circulating.

Recently, in April 2013, PEDV was identified for the first time in the USA, on pig farms located in Ohio [30]. The virus spread quickly within the country and 1 year after the first description, the number of PED affected farms exceeded 5000 spreading to over 25 states. In addition, the virus spread to other countries in North, Central and South America and PED outbreaks were reported for the first time on pig farms in Mexico (July 2013), Peru (October 2013), the Dominican Republic (November 2013), Canada (January 2014), Colombia (March 2014) and Ecuador (July 2014) [31].

### Etiology

Based on genomic analysis, the *Coronaviridae* family has been recently divided into four genera: *Alphacoronavirus, Betacoronavirus, Gammacoronavirus* and *Deltacoronavirus* [32]. PEDV is a member of the genus *Alphacoronavirus* together with other coronaviruses which infect pigs (TGEV and its respiratory variant, porcine respiratory coronavirus or PRCV), dogs (canine coronavirus), cats (feline infectious peritonitis virus), humans (human coronavirus 229E or human coronavirus NL-63) or bats. There are also other swine coronaviruses (Table 1). Porcine hemagglutinating encephalomyelitis virus (PHEV) is a *Betacoronavirus* which causes an infection associated with chronic emaciation and death in young pigs (vomiting and wasting disease) while porcine deltacoronavirus (PDCoV), a member of the genus *Deltacoronavirus*, has recently been identified as the etiological agent of an enteric disease similar to PED or TGE [33].

**Table 1:** Main characteristics and distribution of infections caused by swine coronaviruses

Virus	Genus	Main characteristics of clinical disease	Distribution
Porcine Epidemic Diarrhoea Virus (PEDV)	Alpha	Acute and watery diarrhoea in pigs of all ages.	Only sporadic outbreaks in Europe during the last 10 years but a relevant cause of diarrhoea in pig farms in Asia since the 80s. Firstly described in America in 2013.
		Mortality can reach up to 100 % in suckling piglets of less than 2 weeks due to severe dehydration.	
Transmissible Gastroenteritis Virus (TGEV)	Alpha	Enteric disease clinically indistinguishable of porcine epidemic diarrhoea.	Only very sporadic outbreaks in countries where PRCV is widespread.
Porcine Respiratory Coronavirus (PRCV)	Alpha	Self-limiting respiratory infection. Often subclinical but can exacerbate respiratory symptoms caused by other pathogens.	Endemic infection in many European swine herds.
Hemagglutinating Encephalomyelitis Virus (HEV)	Beta	Neurotropic virus causing the typical vomiting and wasting disease or acute encephalomyelitis with motor disorders in piglets.	Widespread infection although most of the cases remain subclinical.
Porcine Delta Coronavirus (PDCoV)	Delta	Mild to moderate enteric disease in young piglets similar to porcine epidemic diarrhoea or transmissible gastroenteritis.	First identified in Hong Kong, China, in 2009 and North America in early 2014. However, a recent research detected anti-PDCoV IgG antibodies in serum samples collected in 2010, indicating an earlier undetected presence of PDCoV in the US pig population.

Coronaviruses are enveloped viruses which possess a positive-sense single-stranded RNA genome. They are morphologically characterized by the presence of projections or peplomers on their surface. Like other members of the *Alphacoronavirus* genus, PEDV possesses four structural proteins: three membrane proteins identified as S protein or spike protein, M protein or membrane protein and E protein (previously sM or small membrane protein) and a nucleocapsid protein or N protein which encapsidates viral RNA. S protein is particularly relevant among the structural proteins. It is a glycoprotein which induces neutralizing antibodies and interacts with cell receptor in the host. There are also three non-structural proteins: two of them are encoded in open reading frames (ORF) 1a and 1b and are involved in genome replication and transcription while the third, encoded in ORF3, has been reported to be an ion-channel protein [3–5].

Antigenic relationships in PEDV and other coronavirus have been researched into [34]. Although some cross-reactivity between PEDV and TGEV associated with one epitope on the N-terminal region of N protein was recently reported, pig TGEV antisera do not neutralized PEDV and vice versa. No cross-reactivity has been reported between PEDV and any other coronavirus of the beta, gamma or delta genera.

### Infection sources and transmission

Direct and indirect PEDV transmission occurs mainly by faecal-oral route. Viral shedding in faeces starts on post-infection day one or two and continues for a period of 7 to 10 days [35, 36], although it can extend up to 36 weeks in some animals [37, 38]. The transmission of the infection is facilitated by the high viral load in faeces from infected animals [39, 40] as well as by the minimum infectious dose required to infect naïve pigs [31]. Moreover, the resistance of the virus in the environment facilitates the faecal-oral transmission. PEDV is stable under low temperatures, while it is adversely affected by high temperatures. It survives between pH 5.0–9.0 at 4 °C while only between pH 6.5–7.5 at 37 °C. It can survive for at least 28 days in slurry at 4 °C, 7 days in contaminated dry feed at 25 °C or 14 days in contaminated wet feed at 25 °C [31]. This fact favours the indirect transmission by different faeces-contaminated fomites such as transport vehicles [41], feed [42], clothing or footwear.

Genetic and phylogenetic analyses of American PEDV isolates revealed a close relationship with Chinese isolates and their likely Chinese origin [43]. However, how the virus might have travelled from China to the USA is a matter of speculation.

The rapid spread of PEDV on swine farms in the USA raised questions regarding the possibility of airborne transmission of this infection. Although undoubtedly the faecal-oral route is the main source of PEDV transmission, it has been suggested [44] that PEDV may travel through the air for short distances on faecal dust particles, at least under certain conditions. However, airborne transmission of PEDV has only been shown under experimental conditions and up to now infectious PEDV has not been demonstrated in field air samples containing PEDV genetic material [44, 45]. The role that vectors play in the transmission of PEDV has also been investigated. So far, there has been no evidence of PEDV replication in non-porcine hosts, including rodents and starlings [46–48]. However, the potential role of vectors in the mechanic transmission of the virus from one farm to another cannot be ruled out, as has been described for TGEV [4].

Using highly sensitive molecular assays the presence of viral RNA has been reported in milk samples from infected lactating sows [28, 29] as well as in semen samples [29, 31]. However, infectious PEDV in these samples has not been demonstrated and their contamination with faecal material in the sampling cannot be excluded. Moreover, viral RNA has been detected in the serum fraction of whole blood samples from infected pigs [40, 49].

The role of spray-dried porcine plasma (SDPP), normally used as feed additive, as a potential vehicle of transmission of PEDV has been researched into. A number of experimental studies have demonstrated that spray-drying process as well as storage conditions are sufficient to inactivate infectious PEDV in SDPP [50, 51]. The infectivity of commercial SDPP positive for PEDV-RNA has also been investigated. A research group from Canada managed to reproduce PEDV infection in SDPP-inoculated piglets, although they failed to reproduce the infection in animals receiving feed supplemented with the same PEDV-positive SDPP [52]. Similarly, neither clinical signs nor PEDV RNA in faeces or PEDV specific antibodies were detected in pigs which were fed a diet containing 5 % SDPP confirmed positive for PEDV, in a bioassay experiment conducted by Opriessnig et al. [53]. According to this, there is no experimental evidence of PEDV transmission through PCR positive SDPP supplemented feed. This experimental data is corroborated by the fact that despite the use of large amounts of PEDV positive SDPP from the USA to feed pigs in Brazil or Western Canada, these areas remained free of PEDV infection [54].

### Pathogenesis, clinical signs and lesions

PEDV replicates in the cytoplasm of villous enterocytes of the small intestine and causes villous shortening and reduced enzymatic and absorptive capacity in the small intestine causing profuse watery diarrhoea, which lasts about a week [37, 55, 56]. Other clinical signs which are frequently associated to PEDV infection include vomiting, anorexia and fever. Although pigs of all ages are affected, the severity of PED is higher in suckling piglets of less than one week old which may die due to severe dehydration. The slower turnover of enterocytes in neonatal piglets (5–7 days) compared to three weeks-old piglets (2–3 days) could explain, at least partially, the higher susceptibility of these young piglets to PEDV [4].

PEDV has also been detected in epithelial cells of the colon in both experimentally and naturally infected pigs, although villous atrophy has not been demonstrated in the large intestine [40].

Replication of PEDV was classically circumscribed to the intestinal tract [3], until a recent research showed PEDV replication in alveolar macrophages of 3 day-old- colostrum-free piglets, which were experimentally inoculated with a Korean wild-type PEDV isolate [57]. Further studies are needed to confirm whether extra-intestinal replication also occurs with other PEDV isolates as well as to determine their clinical and epidemiological relevance.

Two epidemiologic presentations of PED have been described on the farms. (a) Epidemic PED outbreaks occur when PEDV is introduced into a naïve farm (where most of the animals are PEDV seronegative). The disease spreads rapidly affecting pigs of all ages with morbidity approaching 100 %. Moreover, PEDV can persist and become (b) endemic on the farm affecting post-weaning piglets that have lost their lactogenic immunity as well as newly introduced seronegative gilts.

Mortality associated with PED outbreaks is highly dependent on the age of the infected animals. Mortality can reach up to 80–100 % in suckling piglets of less than one week old, while in weaned pigs mortality rates are typically only 1 to 3 % [11, 30]. No mortality associated with PED is usually observed among adult pigs.

As has already been mentioned, differences in the severity of PED outbreaks have been reported. Particularly severe PED outbreaks have been described in Asia since 2010 and also in the USA. Differences in the virulence of PEDV isolates have been proposed to explain this variability [28, 58, 59]. From our point of view, this is one of the most relevant questions to face regarding PED nowadays: the reason or reasons which could explain variations in the clinical outcome of an outbreak. Although some reports have suggested that they could be associated with differences in the virulence of PEDV isolates, exhaustive challenge studies using pig adapted virus (not cell culture adapted isolates) in suckling piglets are needed to elucidate the role of the strain.

**Fig. 1:**
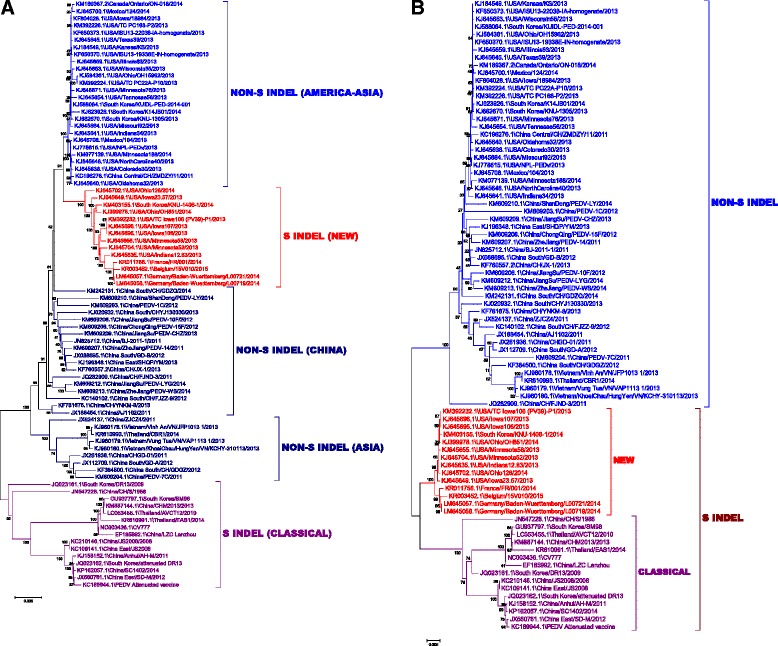
Phylogenetic analysis using the neighbor-joining method based on the nucleotide sequences corresponding to the whole genome (**a**) and full-length S gene (**b**) of a selection of PEDV isolates based on geographical and time criteria. Bootstrap values calculated from 1000 replicate analyses are shown in the nodes

Some insights have been obtained related to the virulence of different strains. In the USA, at least two main variants of PEDV have been recently identified using molecular methods. The first one seems to be a highly virulent virus and similar to viruses described in several Asian countries after 2010 while the second, the S INDEL variant, has been associated to mild clinical outbreaks [59]. This S INDEL variant includes some particular insertions and deletions in the S gene and is also similar to some Asian isolates, part of which were recovered before 2010. The classical European reference strain of PEDV CV777 is also an S INDEL isolate although it is located in a different cluster and well differentiated from American INDEL isolates (Fig. 1a and b). PEDV isolates recovered in European countries (Germany, Italy, Belgium, the Netherlands and France) in 2014 and 2015 have been characterized and all of them were found to be INDEL isolates similar to the variant described in the USA [13–19]. Most of these recent PED outbreaks in Europe occurred in fattening farms and, as expected, no mortality was observed. However, PEDV isolates recently recovered in severe outbreaks of PEDV in Ukraine have shown a genome nucleotide similarity reaching 99.8 % with non-INDEL isolates from the United States and Mexico [20]. So far, this has been the only report of PEDV non-INDEL isolates in Europe. Apart from differences in the virulence of the PEDV strains, many other parameters including management, immune status of the population and herd sanitary status could also explain variations in the clinical outcome of PED outbreaks [31]. Thus, the contribution of co-infections with other viruses, particularly with other enteric viruses such as porcine delta coronavirus (PDCoV) or the recently described mammalian orthoreovirus 3 (MRV3) has also been pointed out. Both viruses have been detected in faecal samples collected from PEDV positive farms in the USA. PDCoV has been associated with mild to moderate diarrhoea in experimentally inoculated naïve suckling piglets [33] while MRV3 caused severe diarrhoea with 100 % mortality in 3-day-old piglets [60].

### Diagnosis

Although the rapid spread of a disease characterized by profuse watery diarrhoea affecting pigs of all ages allows the clinician to suspect that a viral agent is involved in the infection, differential diagnosis to identify PEDV at the laboratory would be needed.

Direct detection of PEDV in faecal samples by conventional or real-time PCR, are the most frequent assays used at present [27]. PCR-assays are generally based on the amplification of fragments within the M, N or S protein genes and are associated with a high sensitivity and specificity. There are also some ELISAs, which are usually based on the use of monoclonal antibodies against PEDV. Although their analytical sensitivity is generally lower than PCR assays [61], they are useful under field conditions as the amount of virus in faecal samples from diseased animals in epidemic outbreaks of PED is very high.

Immunohistochemistry (IHC) is also a very useful tool based on the detection of PEDV antigens within infected cells in formalin-fixed sections of small intestine. It is less sensitive than molecular diagnostic methods but, in contrast, it allows for the evaluation of tissue lesions [62]. In order to increase the sensitivity of IHC assays, several sections of the small intestine of affected pigs sacrificed in the acute phase of the infection should be investigated.

Indirect methods are focused on the detection of antibodies. The detection of PEDV specific antibodies is very useful, not for the investigation of diarrhoea outbreaks, but to determine whether an animal or a herd has previously been infected by this virus. Taking this approach into account, serology is a good tool for surveillance as it provides useful information regarding viral spread in a region or a country. However, the number of tests for the detection of PEDV specific antibodies is limited to ELISAs, indirect immunofluorescence assays (IFA), immunoperoxidase monolayer assays (IPMA) and seroneutralization. Most of these tests are in-house assays and information regarding their sensitivity and specificity is usually scarce. In general, the ELISA tests have proven to be capable of detecting PEDV specific antibodies a little earlier and for longer periods of time than IFA tests [35].

### Control and prevention

There is no specific treatment for PEDV other than supportive care and symptomatic treatment. Mortality occurs in suckling piglets as a result of dehydration which should be corrected using oral electrolyte solutions. In adult pigs, dry feed should be withdrawn for a period of 12–24 h and then, carefully reintroduced while water should be kept freely available [3, 4]. In order to increase passive immunity to piglets and minimize losses, sows due to farrow in at least 2 weeks can be deliberately exposed to virulent virus by the oral route. A recent study revealed that morbidity was reduced from 100 to 43 % in litters exposed to virulent PEDV when their sows were previously exposed to a mild virulent strain (S INDEL variant) of PEDV [63]. Oral administration of chicken egg-yolk or cow colostrum containing PEDV immunoglobulins could offer an immunoprophilactic defence [64, 65]. The increase in lactogenic immunity is also the aim of PEDV vaccines which are used in pregnant sows. Attenuated or killed vaccines against PEDV have been used in several Asian countries for years [66]. However, it has been suggested that live vaccines can revert to virulence and their use and usefulness under field conditions have been questioned [5, 27, 67]. Recently, a PEDV subunit vaccine based on the S protein gene of PEDV as well as a vaccine with killed virus have been licensed in the USA [68], although there are still no studies which prove their efficacy. However, PEDV vaccines have never been used in Europe as the disease was not of sufficient economic importance in this area. In general, PEDV vaccines have been reported to be useful to booster antibody response in animals that have already been infected by PEDV.

As there are no specific treatments for the control and potential eradication of the disease from the herd, preventive measures which preclude the introduction of the virus or new PEDV strains in the area, country or farm are of paramount importance. Supported by the detection methods mentioned in the diagnosis, surveillance should be used to certify that trading of swine or related derivatives do not cause the spread of new strains of the virus. Lorries used in transport have been highlighted as a relevant source of transmission [41] and special attention should be paid in the effectiveness of the cleaning and disinfecting protocols to inactivate and remove the virus. At herd level, basic external biosecurity rules such as quarantine of reposition, ban the entrance of unwashed vehicles, strict visitor policies (time interval between visiting two farms, provide footwear and appropriate clothing, showers and so on) should be carried out without exception and internal biosecurity such as controlling the slurry level, carcasses disposal and carcass bin cleaning, movement of the caretakers on the farm and so on could prevent the establishment of an endemic form of the disease. Finally, many virucidal disinfectants have been shown to be effective in inactivating PEDV. Phenol, quaternary ammonium compounds, glutaraldehyde and bleach are examples of such disinfectants. Water temperature is a crucial factor and temperatures over 60 °C help to inactivate the virus. Proper cleaning and disinfecting of facilities and equipment is crucial to control PEDV.

## Conclusions

The emergence and spread of PEDV on US pig farms has aroused growing interest in this coronavirus. The main areas of recent research on this disease have been focused on the molecular characterization of the isolates as well as the sources of infection and means of transmission. Despite the fact that relevant knowledge has increased, there are still a number of questions to be answered. On one hand, any difference in virulence among the PEDV variants described needs to be clarified. On the other hand, the rapid spread of this virus in the USA has raised concerns about its transmission mechanisms. PEDV is mainly spreads by the faecal-oral route either by direct or indirect contact (feed or fomites such as vehicles). Other routes or sources for its transmission such as air-transmission, vectors or SDPP have been investigated although their implication has not been clearly demonstrated.

The recent PED outbreak in the American continent also shows that more research is needed for the control of the disease, based on the development of useful vaccines and surveillance of the virus, standardising its detection in laboratories with the final goal being the limiting of its spread.

## Abbreviations

PED: Porcine epidemic diarrhoea
PEDV: Porcine epidemic diarrhoea virus
TGEV: Transmissible gastroenteritis virus
PRCV: Porcine respiratory coronavirus
PHEV: Porcine hemagglutinating encephalomyelitis virus
PDCoV: Porcine deltacoronavirus
RNA: Ribonucleic acid
ORF: Open reading frame
SDPP: Spray-dried porcine plasma
MRV3: Mammalian orthoreovirus 3
PCR: Polymerase chain reaction
IHC: Immunohistochemistry
IFA: Indirect immunofluorescence assay
